# Cellular immune response of SARS-CoV-2 vaccination in kidney transplant recipients: a systematic review and meta-analysis

**DOI:** 10.3389/fimmu.2023.1220148

**Published:** 2023-07-26

**Authors:** Suwasin Udomkarnjananun, Sivaporn Gatechompol, Asada Leelahavanichkul, Stephen J. Kerr

**Affiliations:** ^1^ Division of Nephrology, Department of Medicine, Faculty of Medicine, Chulalongkorn University and King Chulalongkorn Memorial Hospital, Bangkok, Thailand; ^2^ Excellence Center for Organ Transplantation (ECOT), King Chulalongkorn Memorial Hospital, Thai Red Cross Society, Bangkok, Thailand; ^3^ Renal Immunology and Transplantation Research Unit, Faculty of Medicine, Chulalongkorn University, Bangkok, Thailand; ^4^ HIV-NAT, Thai Red Cross AIDS Research Centre, Bangkok, Thailand; ^5^ Center of Excellence on Translational Research in Inflammation and Immunology (CETRII), Department of Microbiology, Chulalongkorn University, Bangkok, Thailand; ^6^ Immunology Unit, Department of Microbiology, Chulalongkorn University, Bangkok, Thailand; ^7^ Biostatistics Excellence Centre, Research Affairs, Faculty of Medicine, Chulalongkorn University, Bangkok, Thailand; ^8^ The Kirby Institute, University of New South Wales, Sydney, NSW, Australia

**Keywords:** cellular immune response, COVID-19, Elispot, flow cytometry, interferon-γ, kidney transplantation, SARS-CoV-2 vaccine, T cells

## Abstract

**Background:**

Evidence has demonstrated inferior humoral immune responses after SARS-CoV-2 vaccination in kidney transplant recipients compared to the general population. However, data on cellular immune responses in this population have not been established.

**Methods:**

We searched the MEDLINE, Scopus, and Cochrane databases and included studies reporting cellular immune response rates in kidney transplant recipients after receiving SARS-CoV-2 vaccines. Studies that reported factors associated with cellular immune responders or non-responders were also included (PROSPERO: CRD42022375544).

**Results:**

From a total of 1,494 articles searched, 53 articles were included in the meta-analysis. In all, 21 studies assessed cellular immune response by interferon-γ enzyme-linked immunosorbent spot (IFN-γ ELISPOT), 22 studies used interferon-γ release assay (IGRA), and 10 studies used flow cytometric analysis. The pooled response rate after two doses (standard regimen) and three doses of vaccination was 47.5% (95%CI 38.4-56.7%) and 69.1% (95%CI 56.3-80.6%) from IFN-γ ELISPOT, 25.8% (95%CI 19.7-32.4%) and 14.7% (95%CI 8.5-22.2%) from IGRA, and 73.7% (95%CI 55.2-88.8%) and 86.5% (95%CI 75.3-94.9%) from flow cytometry, respectively. Recipients with seroconversion were associated with a higher chance of having cellular immune response (OR 2.58; 95%CI 1.89-3.54). Cellular immune response in kidney transplant recipients was lower than in dialysis patients (OR 0.24; 95%CI 0.16-0.34) and the general population (OR 0.10; 95%CI 0.07-0.14). Age and immunosuppressants containing tacrolimus or corticosteroid were associated with inferior cellular immune response.

**Conclusion:**

Cellular immune response after SARS-CoV-2 vaccination in kidney transplant recipients was lower than in dialysis patients and the general population. Age, tacrolimus, and corticosteroid were associated with poor response. Cellular immune response should also be prioritized in vaccination studies.

**Systematic review registration:**

https://www.crd.york.ac.uk/prospero/, identifier CRD42022375544.

## Introduction

Mortality after infection with coronavirus disease (COVID-19) in kidney transplant recipients is higher compared to the general population (1). Immunosuppressive medications used in transplant recipients blunt the immune response against the SARS-CoV-2 vaccine, thereby increasing the risk of severe COVID-19. Despite global inequities in vaccine availability, COVID-19 vaccines are available in most settings, but transplant recipients are still at risk of severe disease and death because of the insufficient immune response after vaccination (2, 3).

To date, many studies and meta-analyses have demonstrated that humoral immune responses against SARS-CoV-2 vaccines, defined by the presence of anti-spike protein antibodies or neutralizing antibodies, are poor in kidney transplant recipients (4, 5). Factors associated with decreased antibody responses include older age, deceased donor transplantation, antimetabolite use, and recent rituximab or anti-thymocyte globulin use (5). However, protective immunity against SARS-CoV-2 does not depend on humoral immune responses alone but also requires a robust cell-mediated immune response to clear the virus and enhance humoral immune system function (6–8). The current evidence demonstrates that while the humoral immune response is particularly important for blocking SARS-CoV-2 infection, cellular immunity is of relatively greater importance for the prevention of severe disease, hospitalization, and death (9). Both neutralizing antibody and S2-specific interferon-γ T-cell responses protect against breakthrough SARS-CoV-2 infection after vaccination in kidney transplant recipients (10).

Although both humoral and cellular immune response are crucial for viral clearance and protection against COVID-19, only a limited number of studies have reported data on cellular immune responses. Most COVID-19 vaccination studies in kidney transplant recipients have described only seroconversion rates or neutralizing antibody concentrations without providing details regarding the cellular immune response. This systematic review and meta-analysis was conducted to summarize the current evidence on the cellular immune response after SARS-CoV-2 vaccination in kidney transplant recipients. Cellular immune response rates after COVID-19 vaccines were compared with humoral responses, and the factors associated with cellular immune response were explored.

## Methods

### Data source and searches

This systematic review was conducted based on the Preferred Reporting Items for Systematic Reviews and Meta-Analyses (PRISMA 2020) Statement (11). Electronic databases, including MEDLINE, Scopus, and Cochrane Central Register of Controlled Trials, were searched for eligible studies published in English up to 7 May 2023. The search strategy for MEDLINE using Medical Subject Headings (MeSH) was as followed: (“COVID-19 Vaccines” [MeSH]) AND (“Kidney Transplantation” [MeSH]). The search term in Cochrane Central Register of Controlled Trials included COVID and vaccine and kidney transplantation, exploding all trees of MeSH descriptors. For Scopus, the search strategy was TITLE-ABS-KEY (COVID AND vaccine AND kidney AND transplant). We also reviewed the reference lists in the qualified articles and manually included relevant articles. The protocol for systematic review and meta-analysis was registered in PROSPERO (CRD42022375544).

### Study selection

This primary aim of this systematic review and meta-analysis was to explore cellular immune responses against SARS-CoV-2 vaccination and to determine factors associated with cell-mediated immunity responses in kidney transplant recipients. The included studies were required to report cellular immune response rates, determined by the proportion of patients with cellular immune responses above the specific cutoff used in individual studies, after stimulating with entire spike protein or the S1 subunit. Since cellular immune responses can be evaluated using different assays, including the interferon-γ enzyme-linked immunosorbent spot (IFN-γ ELISPOT), interferon-γ release assay (IGRA), and flow cytometric analysis, studies that presented only the absolute assay results without referencing the positive or negative cellular response rates were excluded from the final analyses. Studies that reported only antibody response were excluded. Evidence regarding factors associated with cellular response was derived from studies assessing associations between potential risk factors in cellular responders and non-responders. We only included studies that reported cellular immune responses in kidney transplant recipients, whether a comparator group was included or not. Two authors (S.U. and S.K.) independently screened the titles and abstracts of the articles and extracted data from full-text articles using a custom-designed spreadsheet. Disagreements were resolved through consensus by all the coauthors.

### Data extraction and quality assessment

The following information was extracted from each study: author names, journal, month and year of article submission (or publication), country of origin, type of COVID-19 vaccine, dose of vaccination, cellular immune response assays and their positive cutoff values, number of kidney transplant recipients included for the evaluation of cellular immune responses, results of cellular immune responses, time of assessment post-vaccination, antibody responses (if presented), cellular immune responses in dialysis patients or (healthy) control population (if presented), and the characteristics of kidney transplant recipients between responders or non-responders. Quality assessment was conducted by using the Newcastle–Ottawa scale (12), which is categorized into three domains: selection, comparability, and outcome. Total scores of 0-3 were considered poor quality, 4-6 fair quality, and 7-9 were considered good quality studies.

### Data synthesis and analysis

Random-effects model meta-analysis was used to 1) calculate the pooled cellular immune response rates following different numbers of vaccine doses and assess cellular immune responses by seroconversion status, 2) to compare cellular immune responses between kidney transplant recipients and other populations and the factors associated with being the responders. The timing of evaluation was considered post-vaccination if the evaluation was performed within 1-8 weeks after receiving the vaccines. Studies that reported the immune response more than 12 weeks after the previous dose (and before the next dose) were reported separately from those reported in the immediate post-vaccination category. If available, antibody and cellular response data were compared and categorized into E+A+ (both cellular and antibody response), E+A- (cellular response without antibody response), E-A+ (antibody response without cellular response), and E-A- (lack of both cellular and antibody response). Pooled odds ratios (ORs) were calculated using the logarithm of the effect size and standard error from each study. If study provided both ORs and adjusted ORs for the cellular immune response, the adjusted ORs were to be used for this analysis. All pooled estimates were provided with a 95% confidence interval (95%CI). Heterogeneity of the pooled effect sizes was evaluated by the *I^2^* index and Q-test p-value. An *I^2^* index higher than 75% implies medium to high heterogeneity. Small-study effect was assessed by Egger’s test. Funnel plots were used to graphically assess the possibility of publication bias of the included studies. Skewed or asymmetric scatter plots of the pooled effect estimates relative to the standard error as a measure of study size indicated possible publication bias or other biases (13). The analyses were performed using Stata 17.0 (StataCorp LLC, College Station, TX) and GraphPad Prism 9.4.0 (GraphPad Software, San Diego, CA).

### Role of the funding sources

The funder of the study had no role in study design, data collection, data analysis, data interpretation, or writing of the report.

## Results

### Characteristics of included studies

**Figure 1:**
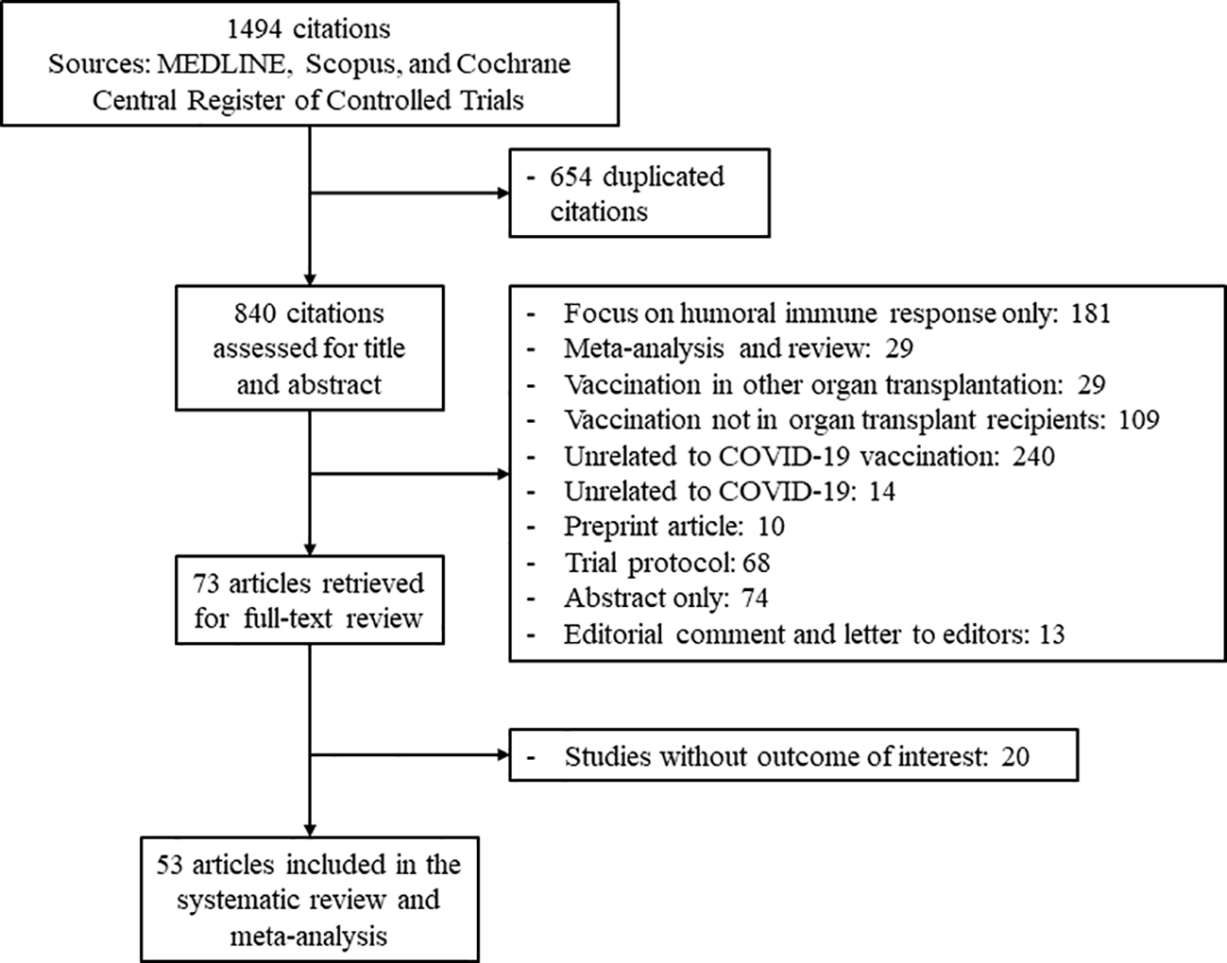
Flow diagram of study selection.

The study flow diagram is shown in **Figure 1**. From a total of 1,494 citations retrieved based on our criteria, 53 articles reporting outcomes on 3,138 transplant recipients were included in the analyses (14–66). In all, 28 studies examined cellular immune responses after two doses of SARS-CoV-2 vaccine, and 25 studies evaluated the response of booster (third or fourth) doses. The characteristics of each study are presented in **Table 1**. Immune responses after mRNA vaccination were evaluated in 50 studies, and 12 studies investigated the response after viral vector vaccination. The inactivated vaccine and protein subunit vaccine were utilized in one study each. Spike protein antigen from SARS-CoV-2 was used to stimulate cellular immune responses after vaccination. A total of 21 studies assessed this cellular response by the IFN-γ ELISPOT assay, 22 studies used IGRA to identify responders and non-responders, and 10 studies explored the cellular immune response using flow cytometric analysis with intracellular cytokine and/or surface antigen staining. **Supplementary Table S1** shows the quality assessment of the included studies.

**Table 1:** Summary of included studies.

Reference	Authors	Journal	Submission to journal (or first published if data were not available)	Country	Total vaccine dose	Type of vaccine	Cellular immune response testing against spike protein	Cutoff	Number of transplant recipients included	Testing time after vaccination (weeks)	Excluded recipients with previous infection	Reported association between cellular and antibody response in KTR	Cellular immune response tested in dialysis patients	Cellular immune response tested in (healthy) control population
(14)	Affeldt et al.	Microorganisms	2021 November	Germany	2	mRNA	IGRA	0.15 IU/ml	32	8.7	Yes	No	0	0
(15)	Affeldt et al.	Viruses	2022 September	Germany	3-4	mRNA or viral vector vaccine	IGRA	0.15 IU/ml	32	6	Yes	Yes	0	0
(16)	Arias-Cabrales et al.	Transplantation	2022 March	Spain	3	mRNA	IGRA	0.15 IU/ml	65	4	Yes	No	74	31
(17)	Bertrand et al.	Journal of American Society of Nephrology	2021 April	France	2	mRNA	IFN-γ ELISPOT	9-20 SFC/mil T cell	45	4	Yes	No	9	0
(18)	Bertrand et al.	Kidney International	2021 October	France	3	mRNA	IFN-γ ELISPOT	25 SFC/mil T cell	80	4	Yes	Yes	0	0
(19)	Bertrand et al.	American Journal of Transplantation	2022 January	France	3	mRNA	IFN-γ ELISPOT	25 SFC/mil T cell	39	4	Yes	No	0	0
(20)	Boedecker-Lips et al.	Pathogens	2022 January	Germany	2	mRNA	IFN-γ ELISPOT	3-7-fold higher than negative control	25	4	Yes	Yes	8	5
(21)	Bruminhent et al.	Scientific Reports	2021 November	Thailand	3	Viral vector after inactivated vaccine	IFN-γ ELISPOT	6 SFC/mil PBMC	31	2	Yes	No	59	16
(22)	Cassaniti	Vaccines	2022 May	Italy	3	mRNA	IFN-γ ELISPOT	10 SFC/mil PBMC	55	4	Yes	No	0	0
(23)	Charmetant et al.	American Journal of Transplantation	2021 September	France	2	mRNA	IGRA	0.07 IU/mL	76	1.4	Yes	No	0	0
(24)	Chavarot et al.	Transplantation	2021 March	France	2	mRNA	IFN-γ ELISPOT	20 SFC/mil T cell	23	4	Yes	No	0	0
(25)	Chen et al.	Frontiers in Immunology	2022 May	Taiwan	2	Viral vector, mRNA, and protein subunit vaccine	IGRA	100 mIU/mL	154	4	Yes	No	0	0
(26)	Crespo et al.	American Journal of Transplantation	2021 July	Spain	2	mRNA	IGRA	0.015 IU/mL	90	4	Yes	No	87	32
(27)	Cucchiari et al.	American Journal of Transplantation	2021 April	Spain	2	mRNA	IFN-γ ELISPOT	6 SFU/2 x 10^5^ PBMC	117	2	No	Yes	0	0
(28)	Cucchiari et al. naïve	Transplant Direct	2022 August	Spain	3	mRNA	IFN-γ ELISPOT	6 SFU/2 x 10^5^ PBMC	105	4	Yes	No	0	0
(29)	Devresse et al.	Transplantation	2021 June	Belgium	2	mRNA	IGRA	100 mIU/mL	90	4	No	Yes	0	0
(30)	Fernandez-Ruiz et al.	Transplant Direct	2021 July	Spain	2	mRNA	IFN-γ ELISPOT	25 SFU/mil PBMC	42*	2	No	Yes	0	28
(31)	Graninger et al.	Journal of Clinical Virology	2023 February	Germany	2	mRNA	IGRA	100 mIE/mL	71	4	Yes	No	65	20
(32)	Hall et al.	American Journal of Transplantation	2021 May	Canada	2	mRNA	Flow cytometric analysis (IFN-γ and IL-2)	3-SD above background plus a minimal polyfunctional T-cell frequency of 0.01%	48*	4	Yes	Yes	0	0
(33)	Imhof et al.	Transplant Direct	2022 July	Netherlands	3	mRNA	IFN-γ ELISPOT	50 SFU/mil PBMC	20	4	Yes	No	0	0
(34)	Kho et al.	Lancet Infectious Diseases	2022 October	Netherlands	3	mRNA or viral vector booster after mRNA vaccine	IFN-γ ELISPOT	50 SFU/mil PBMC	111	4	Yes	No	0	0
(35)	Korber et al.	Frontiers in Immunology	2023 February	Germany	3	mRNA or viral vector vaccine	ELISPOT	2-fold higher than unstimulated control	18	3	Yes	No	0	0
(36)	La Milla et al.	Clinical Kidney Journal	2021 December	Italy	2	mRNA	IGRA	0.15-0.20 IU/mL	18	12	Yes	No	0	18
(37)	Magicova et al.	Transplantation	2021 September	Czechia	2	mRNA	IGRA	0.15 IU/mL	50	2	No	Yes	0	0
(38)	Netti et al.	American Journal of Transplantation	2021 November	Italy	2	mRNA	IGRA	N/A	40	4	Yes	No	0	0
(39)	Panizo et al.	Clinical Kidney Journal	2021 December	Spain	2	mRNA	Flow cytometric analysis (IFN-γ)	Frequency value of SARS-CoV-2-reactive IFN-γ producing CD4+ or CD8+ T cells after background subtraction	26	2	Yes	No	64	15
(40)	Perez-Flores et al.	Frontiers in Immunology	2022 November	Spain	3	mRNA	Flow cytometric analysis (IFN-γ)	0.05%	144	8	Yes	es	18	23
(41)	Piotrowska et al.	Frontiers in Immunology	2021 December	Poland	2	mRNA	IGRA	above background	30	3	No	Yes	56	34
(42)	Prendecki et al.	Lancet	2021 October	United Kingdom	2	mRNA or viral vector vaccine	IFN-γ ELISPOT	40 SFU/mil PBMC	79	4	Yes	Yes	0	32
(43)	Reindl-Schwaigh et al.	JAMA Internal Medicine	2021 November	Austria	3	mRNA or viral vector booster after mRNA vaccine	IGRA	0.1 IU/mL	197	4.2	Yes	No	0	0
(44)	Reischig et al.	American Journal of Transplantation	2021 August	Czechia	2	mRNA	IFN-γ ELISPOT	11-13 SFC/2 x 10^5^ PBMC	31	4	Yes	Yes	0	0
(45)	Rezahosseini et al.	Frontiers in Immunology	2022 October	Denmark	3	mRNA	IGRA	200 mIU/mL	93	18	No	No	0	0
(46)	Sanders et al.	Transplantation	2021 August	Netherlands	2	mRNA	IGRA	0.149 IU/mL	68	4	Yes	Yes	42	46
(47)	Sanders et al.	Clinical Infectious Diseases	2022 April	Netherlands	2	mRNA	IGRA	0.15 IU/ml	62	4	Yes	No	38	40
(48)	Sattler et al.	Journal of Clinical Investigation	2021 April	Germany	3	mRNA	Flow cytometric analysis (CD154/CD137-positive CD4)	2-fold higher frequencies than unstimulated control	39	1.1	Yes	No	26	39
(49)	Sattler et al.	Transplant International	2022 May	Germany	2	mRNA	Flow cytometric analysis (CD154/CD137-positive CD4)	2-fold higher frequencies than unstimulated control	20	5.6	Yes	No	0	13
(50)	Schmidt et al.	American Journal of Transplantation	2022 May	Germany	2	mRNA or viral vector	Flow cytometric analysis (CD69/IFN-γ-positive CD4 or CD8 cells)	0.03% of reactive T cells	34*	2	Yes	Yes	0	70
(51)	Schrezenmeier et al.	Journal of Clinical Investigation Insight	2021 December	Germany	4	mRNA booster after mRNA or viral vector vaccine	Flow cytometric analysis (CD154/CD137-positive CD4)	3-fold higher frequencies than unstimulated control	27	1	Yes	No	0	0
(52)	Schrezenmeier et al.	Journal of American Society of Nephrology	2021 July	Germany	3	mRNA or viral vector booster after mRNA vaccine	Flow cytometric analysis (CD154/CD137-positive CD4)	2-fold higher frequencies than unstimulated control	25	1	Yes	No	0	0
(53)	Stumpf et al.	Frontiers in Medicine	2022 April	Germany	3	mRNA or viral vector booster after mRNA vaccine	IGRA	100 mIU/mL	71	4	Yes	No	0	0
(54)	Stumpf et al.	Lancet Regional Health	2021 May	Germany	2	mRNA	IGRA	100 mIU/mL	124	4	Yes	Yes	119	35
(55)	Stumpf et al.	Frontiers in Medicine	2022 April	Germany	2	mRNA	IGRA	100 mIU/mL	42	20	Yes	No	0	0
(56)	Stumpf et al.	Transplantation	2021 June	Germany	3	mRNA	IGRA	100 mIU/mL	35	4	Yes	No	0	0
(57)	Takai et al.	Frontiers in Immunology	2022 September	Japan	3	mRNA	ELISPOT	164 cytokine activity	53	4	Yes	No	0	0
(58)	Takai et al.	International Journal of Urology	2022 April	Japan	2	mRNA	IFN-γ ELISPOT	164 cytokine activity	58	4	Yes	Yes	0	10
(59)	Thomson et al.	eClinicalMedicine	2022 June	United Kingdom	4	mRNA booster after mRNA or viral vector vaccine	IFN-γ ELISPOT	40 SFU/mil PBMC	54	7.1	Yes	Yes	0	0
(60)	Thummler et al.	Vaccines	2022 July	Germany	3	mRNA	IFN-γ ELISPOT	3 SFU/2.5 x 10^5^ PBMC	32	30.3	Yes	No	0	17
(61)	Tometten et al.	Journal of Infectious Diseases	2022 July	Germany	3-4	mRNA or viral vector	ELISPOT	28 SFC/mil	113	15	Yes	No	0	35
(62)	Watcharananan et al.	American Journal of Transplantation	2021 November	Thailand	2	Viral vector	IGRA	200 mIU/mL	67	3.6	Yes	No	0	15
(63)	Westhoff et al.	Kidney International	2021 September	Germany	3	mRNA	Flow cytometric analysis (CD154/CD137-positive CD4)	0.01% of CD4 or CD8 T cells	10	2	Yes	Yes	0	0
(64)	Yahav et al.	Transplant International	2021 November	Israel	3	mRNA	IGRA	10 pg/mL	53	3	Yes	No	0	0
(65)	Zhang et al.	Frontiers in Immunology	2022 September	China	3	Inactivated	ELISPOT	69.09 SFC/mil	36	3	Yes	No	0	26
(66)	Zhang et al.	Transplant Infectious Disease	2021 November	United States	2	mRNA	Flow cytometric analysis (IL-2/TNF-α-positive CD4 or TNF-α/IFN-γ-positive CD8 cells)	0.05% of CD4 or CD8 T cells	38	4	Yes	Yes	0	41

ELISPOT; enzyme-linked immunosorbent spot, IFN-γ; interferon-γ, IGRA; interferon-γ release assay; IL-2; interleukine-2, KTR; kidney transplant recipient, PBMC; peripheral blood mononuclear cell, SFC/mil; spot-forming unit per million cells, TNF-α tumor necrosis factor-α.

*Included recipients of other solid organ transplantation because the analyses of only kidney transplant recipients were not presented.

### Cellular immune response after SARS-CoV-2 vaccination in kidney transplant recipients

**Figure 2:**
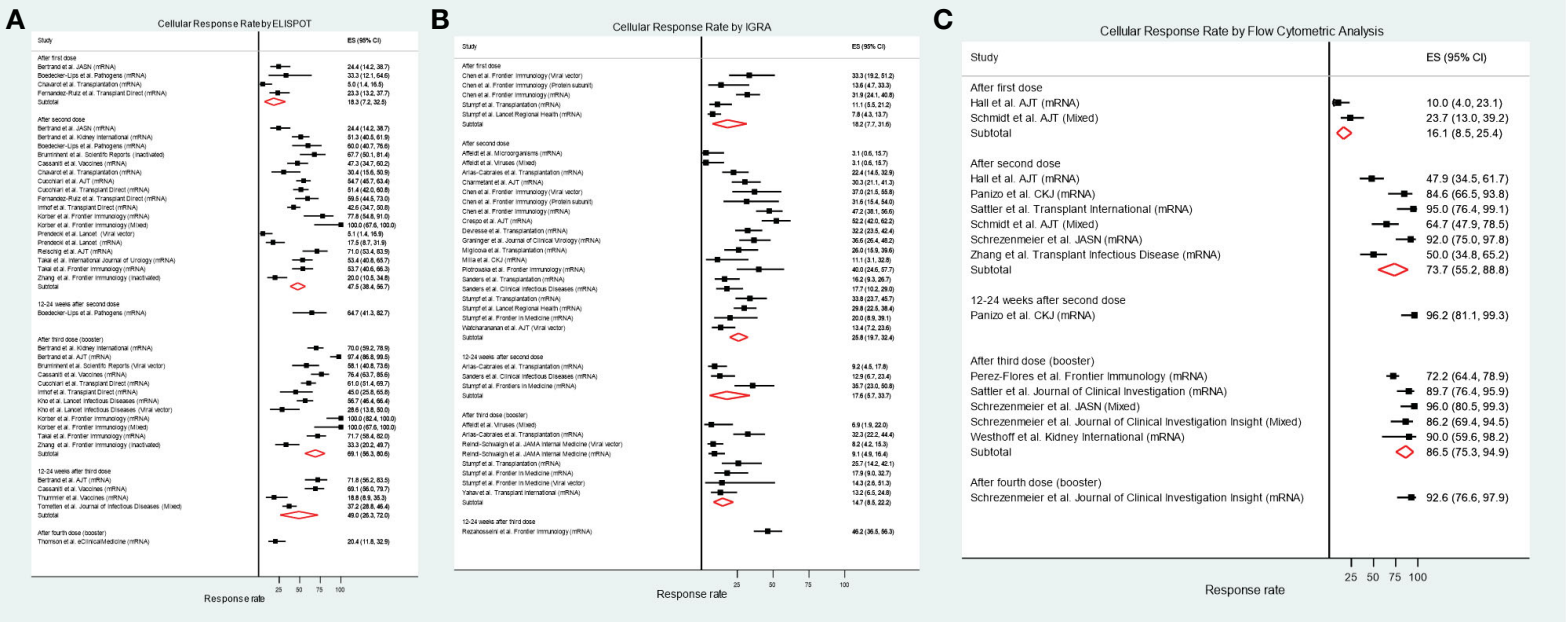
Cellular immune response rate in kidney transplant recipients after receiving different numbers of SARS-CoV-2 vaccine doses. **(A)** IFN-γ ELSIPOT assay. **(B)** IGRA. **(C)** Flow cytometric analysis.

**Figure 2** shows the cellular response rates by the different testing methods. For studies that used IFN-γ ELISPOT assays, the pooled response rate after the first vaccine dose was 18.3% (95%CI 7.2-32.5%; *I^2^* 68.3%), after the second dose it was 47.5% (95%CI 38.4-56.7%; *I^2^* 86.5%), and after a third booster dose it was 69.1% (95%CI 56.3-80.6%; *I^2^* 88.5%) (**Figure 2A**). Pooled cellular immune response rates assessed by using IGRA were 18.2% (95%CI 7.7-31.6%, *I^2^* 86.8%) after the first dose, 25.8% (95%CI 19.7-32.4%; *I^2^* 82.1%) after the second dose, and 14.7% (95%CI 8.5-22.2%; *I^2^* 69.6%) after a third booster dose (**Figure 2B**). Studies presenting results from flow cytometric analysis demonstrated pooled response rates of 16.1% (95%CI 8.5-25.4%) after the first dose, 73.7% (95%CI 55.2-88.8%; *I^2^* 85.6%) after the second dose, and 86.5% (95%CI 75.3-94.9%; *I^2^* 70.1%) after a third booster dose (**Figure 2C**). Only six studies included recipients with previous SARS-CoV-2 infection (27, 29, 30, 37, 41, 45). **Supplementary Figure S1** illustrates the cellular immune response rate after excluding the results from these studies, and the results were not different from the primary analyses that included recipients with previous SARS-CoV-2 infection. Some studies that evaluated the response after a third booster dose included only kidney transplant recipients who did not achieve seroconversion after the second dose; these included 3/12 estimates (33, 34) using the IFN-γ ELISPOT assay, 5/8 estimates (43, 53, 56) using the IGRA test, and 3/5 estimates (51, 52, 63) using flow cytometric analysis. **Supplementary Figure S2** shows the cellular immune response after excluding these studies, in which the response rates were comparable to the primary analyses that included all studies. The pooled seroconversion rates based on anti-spike IgG production are shown in **Supplementary Figure S3**. **Supplementary Figure S4** demonstrates funnel plots of the overall cellular and antibody responses of all the included studies. The plots are predominantly symmetrical except the studies using flow cytometric analysis, suggesting possible publication bias or heterogeneity among different flow cytometry techniques.

**Figure 3:**
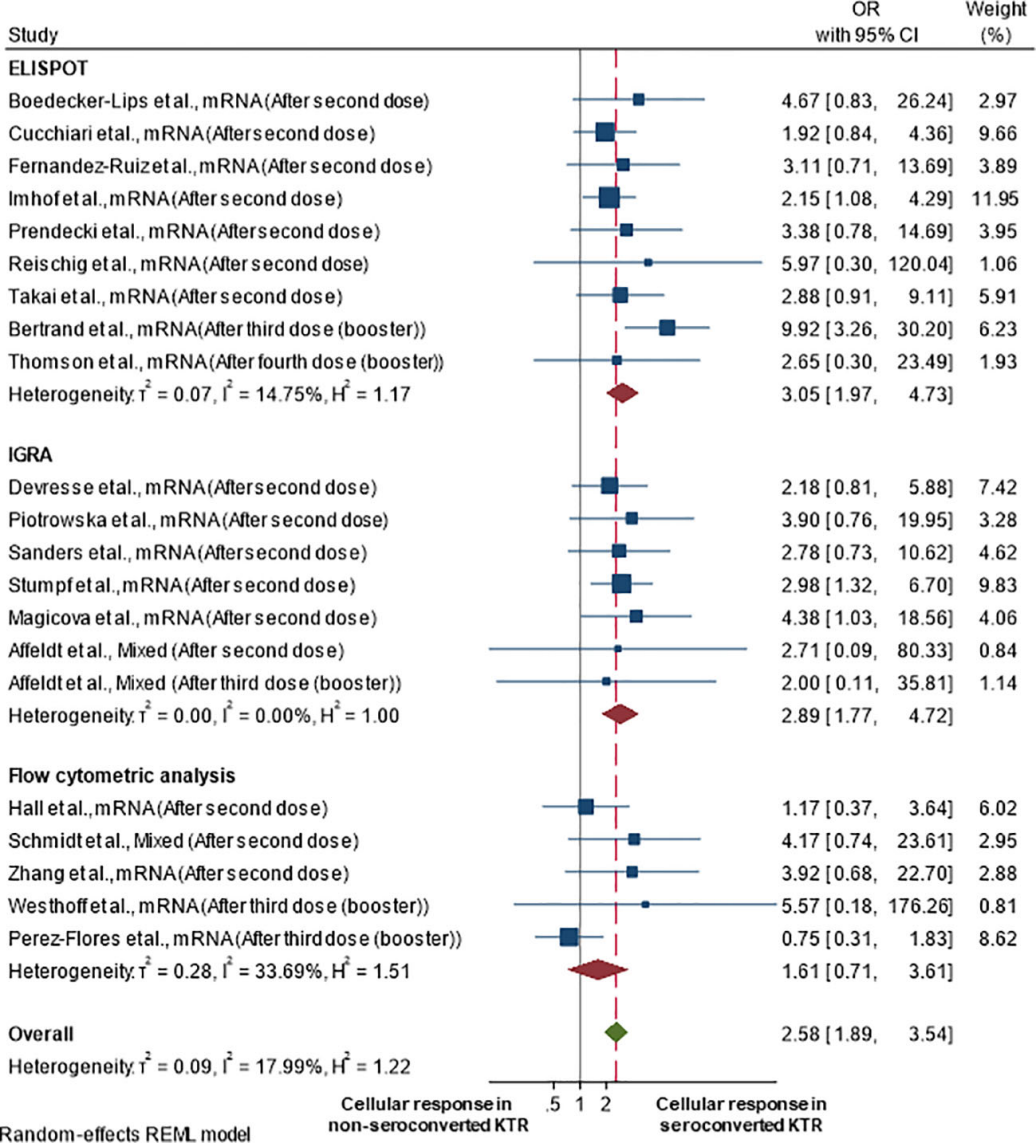
Cellular immune response rate in kidney transplant recipients by seroconversion status after at least two vaccine doses.

**Figure 3** illustrates a forest plot of the cellular immune response in kidney transplant recipients who seroconverted compared to those who did not seroconvert. Recipients who seroconverted were more likely to achieve cellular immune responses (pooled OR 2.58; 95%CI 1.89-3.54; 95% prediction interval 1.26-5.28; p-value<0.001; *I^2^* 18.0%; Q-test 0.569; Egger’s test 0.161) than recipients who failed to seroconvert. This also means that recipients with cellular immune responses had 2.58-fold higher odds of having seroconverted compared with recipients without cellular immune response. A funnel plot of this analysis is shown in **Supplementary Figure S5**, showing some evidence of asymmetry, which may be due to publication bias.

### Comparison of cellular immune response between types of SARS-CoV-2 vaccination

Four studies directly compared cellular immune responses in kidney transplant recipients vaccinated with mRNA versus the viral vector vaccine (34, 42, 43, 53). Although comparison was not significant at <0.05, the pooled effect sizes and 95%CI for both two- and three-dose regimens demonstrated higher cellular immune response rates in kidney transplant recipients who received mRNA versus viral vector vaccines (**Supplementary Figure S6**). Three studies performed head-to-head comparisons of BNT162b2 and mRNA-1273, two after a single dose and a complete primary vaccination series (25, 31) and one after a booster dose (53), which did not show a significant difference of cellular immune responses between these mRNA vaccines.

### Comparison of cellular immune response rates between kidney transplant recipients and other populations

**Figure 4:**
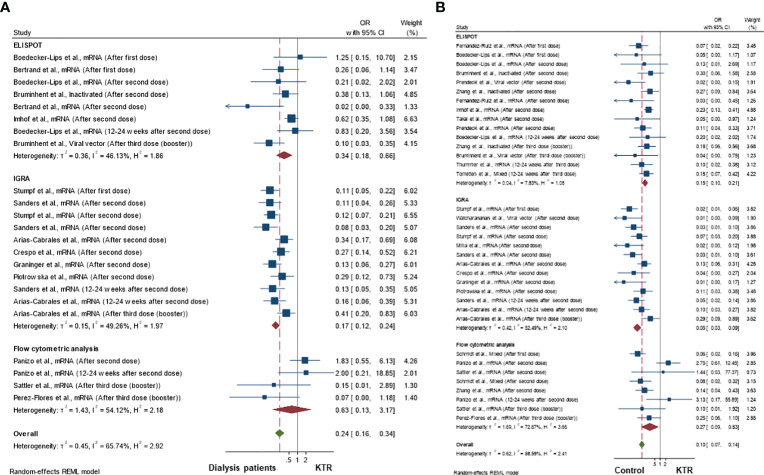
Cellular immune response rates in kidney transplant recipients compared to **(A)** dialysis patients and **(B)** healthy controls population.

**Figures 4A, B** demonstrate the OR of achieving a cellular immune response in kidney transplant recipients compared to dialysis patients or control (non-kidney disease) populations, respectively. Being a kidney transplant recipient was associated with lower odds of being a cellular immune responder compared to both dialysis patients (pooled OR 0.24; 95%CI 0.16-0.34; 95% prediction interval 0.06-1.01; p-value<0.001; *I^2^* 65.7%; Q-test<0.001; Egger’s test 0.869) or controls without kidney disease (pooled OR 0.10; 95%CI 0.07-0.14; 95% prediction interval 0.02-0.51; *I^2^* 58.6%; p-value<0.001; Q-test<0.001; Egger’s test 0.629). The funnel plots presented in **Supplementary Figure S7** appear to be symmetrical, suggesting no publication bias.

**Figure 5:**
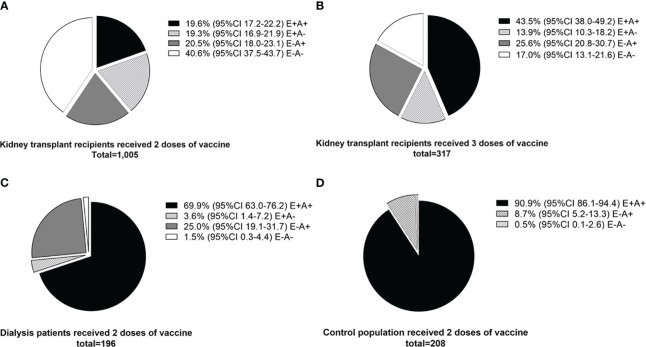
Comparison of cellular and antibody immune responses between different populations. **(A)** Kidney transplant recipients who received two doses of vaccine (standard regimen). **(B)** Kidney transplant recipients who received three doses of vaccine (after booster dose). **(C)** Dialysis patients who received two doses of vaccine. **(D)** Control population who received two doses of vaccine. E+A+; both cellular and antibody response, E+A-; cellular response but no antibody response, E-A+; antibody response but no cellular response, E-A-; both negative cellular and antibody response.

In studies that followed kidney transplant recipients from primary vaccination to boosting doses and categorized them according to their cellular and antibody responses, 19.6 (95%CI 17.2-22.2) % of recipients had both cellular and antibody responses after two doses of vaccination, and this increased to 43.5 (38.0 – 49.2) % after receiving a third booster dose (**Figures 5A, B**). Compared to kidney transplant recipients, dialysis patients and controls were more likely to have both cellular and antibody immune responses after two doses of vaccination (69.9% and 90.9%, respectively) (**Figures 5C, D**).

### Factors associated with cellular immune response in kidney transplant recipients

**Table 2** demonstrates the pooled OR for factors that were evaluated for their association with cellular immune responder in the studies (16–18, 22, 26, 27, 30, 32, 33, 35, 37, 38, 40–42, 45, 50, 55, 57–59, 61, 66). Increasing age was associated with being a cellular immune non-responder (pooled OR 0.98; 95%CI 0.96-1.00; p-value=0.045; I^2^ 36%, Q-test 0.097; Egger’s test 0.043). Tacrolimus (vs. non-tacrolimus regimens) was associated with a reduced odds of being a cellular immune responder (pooled OR 0.53; 95%CI 0.31-0.91; p-value=0.021; I^2^ 51%, Q-test 0.010; Egger’s test 0.091). Corticosteroid-containing regimens were also associated with being non-responders (pooled OR 0.54; 95%CI 0.42-0.70; p-value<0.001; I^2^ 0%, Q-test 0.319; Egger’s test 0.858). Although not reaching statistical significance, the time from transplant showed an association with cellular response rates, with patients less likely to achieve a response if they were <1 year from transplant and more likely to achieve a response as the duration since transplant increased. The forest plots and funnel plots for the factors evaluated in two or more studies for their association with cellular immune responses are illustrated in **Supplementary Figures S8**, **S9**. Many funnel plots show evidence of asymmetry, which might be due to publication bias or the heterogeneity of the included studies, although the interpretation of funnel plots with <10 studies is challenging (67).

**Table 2:** Pooled odds ratios for factors associated with cellular immune responses.

Variable	Pooled odds ratio	95% confidence interval	P-value from random-effects model	95% prediction interval	Number of studies included	*I^2^* index	Q-test p-value	Egger’s test p-value
Age	0.98	(0.96-1.00)	0.045	0.93-1.03	13	36%	0.097	0.043
Female (vs. male)	0.93	(0.74-1.16)	0.504	0.73-1.18	17	0%	0.769	0.849
Tacrolimus (vs. non-tacrolimus regimen)*	0.53	(0.31-0.91)	0.021	0.11-2.47	12	51%	0.010	0.091
Belatacept (vs. non-belatacept regimen)	0.52	(0.11-2.47)	0.410	0.01-412.86	4	70%	0.025	0.077
Mycophenolate (vs. non-mycophenolate regimen)	0.90	(0.69-1.17)	0.419	0.67-1.20	14	0%	0.698	0.570
mTORi (vs. non-mTORi regimen)	1.05	(0.62-1.77)	0.858	0.34-3.27	10	26%	0.180	0.263
Steroid (vs. non-steroid regimen)	0.54	(0.42-0.70)	< 0.001	0.40-0.73	10	0%	0.319	0.858
Diabetes mellitus	0.75	(0.53-1.07)	0.110	0.40-1.42	13	13%	0.358	0.510
Lymphopenia	0.56	(0.19-1.61)	0.280	0.01-38.95	4	60%	0.055	0.968
Duration since transplantation <1 year after transplantation (vs. more than ≥1 year)	0.50	(0.25-1.01)	0.053	0.11-2.24	5	14%	0.172	0.883
Time after transplantation (years)	1.01	(1.00-1.02)	0.052	0.99-1.02	7	13%	0.117	0.013

*Three studies (Imhof et al., Perez-Flores et al., and Tometten et al.) assessed calcineurin inhibitors vs. non-calcineurin inhibitor regimen.

## Discussion

To our knowledge, this study is the first systematic review and meta-analysis to examine the cellular immune response in kidney transplant recipients after SARS-CoV-2 vaccination. Although our primary focus was on kidney transplant recipients, we made comparisons of cellular and humoral immune response rates in dialysis patients and controls after vaccination when these groups were included in individual studies. Positive cellular immune responses were more frequently found in the control population or dialysis patients than in kidney transplant recipients. Kidney transplant recipients who experienced seroconversion after vaccination were more likely to have a cellular immune response. Based on the available data, mRNA vaccines were not more likely to elicit a cellular immune response compared to viral vector vaccines. Age and post-transplant immunosuppressive regimens containing tacrolimus or corticosteroids were associated with a decreased chance of being a cellular immune responder.

The evaluation of cell-mediated immunity in kidney transplant recipients has been shown to provide valuable information on identifying recipients at risk of post-transplant complications. For example, the donor-specific IFN-γ ELSIPOT assay can determine recipients with a high risk of acute rejection (68). Kidney transplant recipients with positive CMV and BK virus-specific IFN-γ ELSIPOT assay or IGRA have a lower risk of CMV or BK virus infection (69, 70). For SARS-CoV-2, T-cell responses are needed to generate and maintain levels of high-affinity antibodies (6). T-cell immunity also plays an important role in preventing initial infection and limiting the extent of disease after infection, thus reducing the severity of disease. Cellular sensitization without seroconversion has been described in individuals with mild or asymptomatic COVID-19, which potentially indicates a role for the cellular immune system in clearing early infection and limiting the spread of the virus (71). A generally accepted concept is that high levels of neutralizing antibodies mediate protection from SARS-CoV-2 infection, and T cells and memory B cells help prevent severe disease and hospitalization (7). Importantly, T-cell immunity provides durable protection and can recognize a broad range of SARS-CoV-2 antigens, including those from variants of concern, where specific neutralizing antibody responses are greatly reduced or absent. T-cell responses are less sensitive to the single amino acid mutations seen in these variants, so loss of cross-protective immunity is unlikely (6–8).

Our study shows that different methods of evaluation resulted in different cellular immune response rates. It is important to recognize that there is currently no single best test for assessing cellular immunity. The negative IFN-γ ELISPOT assay or IGRA results do not imply the complete absence of cellular immune responses in the patients, since several cytokines are involved in the T-cells response, including tumor necrosis-α and interleukin-2 (72). A robust panel of tests would ideally be needed to accurately classify patients as immune responders or non-responders. Currently, flow cytometric analysis of the surface and intracellular staining offer simultaneous assessments of multiple aspects of cellular immunity by determining individual cytokine responses and whether naïve, memory, regulatory, or effector cells are involved. However, flow cytometric analysis requires well-trained personnel and still lacks methodological standardization between centers. On the contrary, commercial ELISPOT and IGRA kits are available but cannot capture every aspect of cellular immune responses (8). Future studies should explore the performance of advanced assays such as multiplex polymerase chain reaction or combinations of multiple cellular response assays to provide a more comprehensive assessment of cellular immune response signatures. This multifaceted assay has the potential to facilitate a more complete picture of the cellular immune response against SARS-CoV-2, consequently aiding in the identification of a precise diagnostic test capable of establishing an optimal threshold for immune protection. Such a study comparing different assays with a large number of included participants would also be useful to standardize evaluations.

The clinical outcomes of a COVID-19 infection, such as infectivity rate, severity and duration of symptoms, and mortality, would be the best indicators to determine the true effect of cellular immune responses versus humoral immune responses. Since some kidney transplant recipients in the studies in this meta-analysis uniquely had isolated cellular immune responses (**Figure 5**), this population could be a candidate to determine the optimal protection thresholds of pure cellular immunity. Additionally, assessing the effect of SARS-CoV-2 vaccines on allograft and patient survival would be interesting to explore in preexisting kidney transplant prediction models (73).

Humoral immune responses after SARS-CoV-2 vaccines in kidney transplant recipients were inferior to those observed in dialysis patients or control populations (74). In addition, the vaccination response rates in dialysis patients were lower compared to the general population, similarly to other vaccines that are routinely prescribed in clinical practice (75, 76). Tacrolimus- and corticosteroid-containing regimens were associated with lower cellular immune response rates. Since tacrolimus inhibits calcineurin activity in T cells (77), one could reasonably expect that tacrolimus significantly affects cell-mediated immunity after vaccination. Glucocorticoids have a wide spectrum of immunosuppressive effects, including the induction of apoptosis of T cells and B cells (78), which also influences the post-vaccination immune response. Although the estimates did not reach a significant level, the longer period between kidney transplantation and vaccination showed a trend of being associated with cellular immune responses. The lower net immunosuppression used in the later period after transplantation compared to the earlier period may explain this association. It should be noted that although age and tacrolimus-containing regimens were significant factors for cellular non-responders based on the 95%CI from the random-effect model meta-analysis, their 95% prediction intervals were wider and included the no-effect OR of 1.0. Relatively few studies provided sufficient information to make a comparison according to age and tacrolimus use. Therefore, this uncertainty might be explained by confounding from other individual factors shown to impact cellular immune response, and further studies may help with more precise estimates.

A strength of this systematic review and meta-analysis is the comprehensive analysis of multiple aspects regarding the cellular immune response against SARS-CoV-2 vaccines in kidney transplant recipients. The results from this study could serve as a reference of cellular response to two doses and booster doses of SARS-CoV-2 vaccines. Future studies can compare these results with those of newer generations of the SARS-CoV-2 vaccine.

### Limitations of the study

There are also some limitations in this study. First, the clinical outcomes after vaccination were not reported in most studies, which precludes the comparison of COVID-19 infection rates and their clinical outcomes between cellular immune responders and non-responders. Second, the information on variant-specific cellular immune responses was not reported in most of the studies. Third, although we presented funnel plots for each outcome, these plots and the corresponding Egger tests results are difficult to interpret when the number of studies is low. Although it seems plausible that authors who conducted cellular immune response studies in specialized disease cohorts would publish their results, the possibility of publication bias cannot be completely discounted. Furthermore, there is evidence of asymmetry in multiple funnel plots, indicating possible publication bias or the heterogeneity of the included studies, although in funnel plots of less than 10 studies, it is difficult to differentiate the chance of asymmetry from real asymmetry, and these plots require careful interpretation (67). Some cohorts might not be able to fully exclude individuals with prior SARS-CoV-2 infection before receiving vaccinations, potentially impacting the rate of cellular immune response. Nonetheless, we mitigated this confounding by conducting a sensitivity analysis of the response rate after excluding studies that included recipients with known SARS-CoV-2 infection. Fourth, most of the included studies assessed the cellular immune response in only a proportion of their entire kidney transplant recipient cohorts without describing whether the selected patients were representative of the entire cohort. For this reason, we did not attempt any meta-regression of the response rates against individual cohort characteristics as the results would be confounded by potential selection bias. Finally, there is a potential for bias in assessing the odds of achieving cellular immune responses in kidney transplant recipients who have seroconverted compared to the non-seroconverters. This bias arises from the inclusion of studies that provided cellular immune response rates while excluding studies that solely reported humoral immune responses without reporting cellular response rates.

## Conclusions

In conclusion, the cellular immune response following SARS-CoV-2 vaccine administration in kidney transplant recipients was lower than in dialysis patients and the control population. Recipients who seroconverted had a higher odds of developing cellular immune responses. Increasing age and taking immunosuppressive regimens that included tacrolimus and corticosteroid were associated with being a non-responder. More studies are needed to standardize the assays used for the evaluation of cellular immune responses and correlate the values with clinical outcomes. The new generation of vaccines should not solely aim for the improvement of humoral immunity but also for achieving an adequate cellular immune response.

## Data availability statement

The raw data supporting the conclusions of this article will be made available by the authors, without undue reservation.

## Author contributions

Idea and study design: SU and SK. Study selection: SU and SK. Data collection and analysis: SU and SK. Writing of the article: SU. Manuscript review and edit: SG, AL, and SK. Funding acquisition: SU. All authors have read the manuscript and approved this submission.
