# Supplementary material for: Cellular immune response of SARS-CoV-2 vaccination in kidney transplant recipients: a systematic review and meta-analysis
Source: Front Immunol. 2023 Jul 26;14:1220148. doi: 10.3389/fimmu.2023.1220148 (PMC10415203; doi:10.3389/fimmu.2023.1220148)

**Cellular Immune Response of SARS-CoV-2 Vaccination in Kidney Transplant Recipients: A Systematic Review and Meta-analysis**

Suwasin Udomkarnjananun^1,2,3^, MD, PhD, Sivaporn Gatechompol^4^, MD, Asada Leelahavanichkul^5,6^, MD, PhD, Stephen J Kerr^4,7,8^, PhD

^1^Division of Nephrology, Department of Medicine, Faculty of Medicine, Chulalongkorn University and King Chulalongkorn Memorial Hospital, Bangkok, Thailand

^2^Excellence Center for Organ Transplantation (ECOT), King Chulalongkorn Memorial Hospital, Thai Red Cross Society, Bangkok, Thailand.

^3^Renal Immunology and Transplantation Research Unit, Faculty of Medicine, Chulalongkorn University, Bangkok, Thailand.

^4^HIV-NAT, Thai Red Cross AIDS Research Centre, Bangkok, Thailand.

^5^Center of Excellence on Translational Research in Inflammation and Immunology (CETRII), Department of Microbiology, Chulalongkorn University, Bangkok, Thailand

^6^Immunology Unit, Department of Microbiology, Chulalongkorn University, Bangkok, Thailand

^7^Biostatistics Excellence Centre, Research Affairs, Faculty of Medicine, Chulalongkorn University, Bangkok, Thailand.

^8^The Kirby Institute, University of New South Wales, Sydney, Australia.

**Corresponding author**

Suwasin Udomkarnjananun

Division of Nephrology, Department of Medicine, Faculty of Medicine, Chulalongkorn University and King Chulalongkorn Memorial Hospital

1873, Rama 4 road, Pathumwan, Bangkok, Thailand, 10330

Tel: +6622564000

Fax: +6622526920

suwasin.u@gmail.com

**Supplementary Table S1**: Newcastle-Ottawa Quality assessment of the included studies

| **Reference** | **Authors** | **Journal** | **Submission to journal (or first published if data was not available)** | **Selection** | **Comparability** | **Exposure** | **Total** |
| --- | --- | --- | --- | --- | --- | --- | --- |
| 14 | Affeldt et al. | Microorganisms | 2021 November | 3 | 0 | 3 | 6 |
| 15 | Affeldt et al. | Viruses | 2022 September | 3 | 0 | 3 | 6 |
| 16 | Arias-Cabrales et al. | Transplantation | 2022 March | 4 | 2 | 3 | 9 |
| 17 | Bertrand et al. | Journal of American Society of Nephrology | 2021 April | 4 | 2 | 3 | 9 |
| 18 | Bertrand et al. | Kidney International | 2021 October | 3 | 2 | 3 | 8 |
| 19 | Bertrand et al. | American Journal of Transplantation | 2022 January | 3 | 0 | 3 | 6 |
| 20 | Boedecker-Lips et al. | Pathogens | 2022 January | 4 | 1 | 3 | 8 |
| 21 | Bruminhent et al. | Scientific Reports | 2021 November | 4 | 1 | 3 | 8 |
| 22 | Cassaniti | Vaccines | 2022 May | 3 | 2 | 3 | 8 |
| 23 | Charmetant et al. | American Journal of Transplantation | 2021 September | 3 | 0 | 3 | 6 |
| 24 | Chavarot et al. | Transplantation | 2021 March | 3 | 0 | 3 | 6 |
| 25 | Chen et al. | Frontier in Immunology | 2022 May | 3 | 1 | 3 | 7 |
| 26 | Crespo et al. | American Journal of Transplantation | 2021 July | 4 | 2 | 3 | 9 |
| 27 | Cucchiari et al. | American Journal of Transplantation | 2021 April | 2 | 2 | 3 | 7 |
| 28 | Cucchiari et al. naïve | Transplant Direct | 2022 August | 3 | 0 | 3 | 6 |
| 29 | Devresse et al. | Transplantation | 2021 June | 2 | 0 | 3 | 5 |
| 30 | Fernandez-Ruiz et al. | Transplant Direct | 2021 July | 3 | 2 | 3 | 8 |
| 31 | Graninger et al. | Journal of Clinical Virology | 2023 February | 4 | 1 | 3 | 8 |
| 32 | Hall et al. | American Journal of Transplantation | 2021 May | 3 | 2 | 3 | 8 |
| 33 | Imhof et al. | Transplant Direct | 2022 July | 3 | 2 | 3 | 8 |
| 34 | Kho et al. | Lancet Infectious Diseases | 2022 October | 3 | 1 | 3 | 7 |
| 35 | Korber et al. | Frontier in Immunology | 2023 February | 3 | 2 | 3 | 8 |
| 36 | La Milla et al. | Clinical Kidney Journal | 2021 December | 4 | 1 | 3 | 8 |
| 37 | Magicova et al. | Transplantation | 2021 September | 3 | 2 | 3 | 8 |
| 38 | Netti et al. | American Journal of Transplantation | 2021 November | 3 | 2 | 3 | 8 |
| 39 | Panizo et al. | Clinical Kidney Journal | 2021 December | 4 | 1 | 3 | 8 |
| 40 | Perez-Flores et al. | Frontier in Immunology | 2022 November | 4 | 2 | 3 | 9 |
| 41 | Piotrowska et al. | Frontier Immunology | 2021 December | 3 | 2 | 3 | 8 |
| 42 | Prendecki et al. | Lancet | 2021 October | 4 | 2 | 3 | 9 |
| 43 | Reindl-Schwaigh et al. | JAMA Internal Medicine | 2021 November | 3 | 1 | 3 | 7 |
| 44 | Reischig et al. | American Journal of Transplantation | 2021 August | 3 | 0 | 3 | 6 |
| 45 | Rezahosseini et al. | Frontier in Immunology | 2022 October | 3 | 2 | 3 | 8 |
| 46 | Sanders et al. | Transplantation | 2021 August | 4 | 1 | 3 | 8 |
| 47 | Sanders et al. | Clinical Infectious Diseases | 2022 April | 4 | 1 | 3 | 8 |
| 48 | Sattler et al. | Journal of Clinical Investigation | 2021 April | 4 | 1 | 3 | 8 |
| 49 | Sattler et al. | Transplant International | 2022 May | 4 | 1 | 3 | 8 |
| 50 | Schmidt et al. | American Journal of Transplantation | 2022 May | 4 | 2 | 3 | 9 |
| 51 | Schrezenmeier et al. | Journal of Clinical Investigation Insight | 2021 December | 3 | 0 | 3 | 6 |
| 52 | Schrezenmeier et al. | Journal of American Society of Nephrology | 2021 July | 3 | 0 | 3 | 6 |
| 53 | Stumpf et al. | Frontier in Medicine | 2022 April | 3 | 1 | 3 | 7 |
| 54 | Stumpf et al. | Lancet Regional Health | 2021 May | 4 | 1 | 3 | 8 |
| 55 | Stumpf et al. | Frontiers in Medicine | 2022 April | 3 | 2 | 3 | 8 |
| 56 | Stumpf et al. | Transplantation | 2021 June | 3 | 0 | 3 | 6 |
| 57 | Takai et al. | Frontier in Immunology | 2022 September | 3 | 2 |  |  |
| 58 | Takai et al. | International Journal of Urology | 2022 April | 4 | 2 | 3 | 9 |
| 59 | Thomson et al. | eClinicalMedicine | 2022 June | 3 | 2 | 3 | 8 |
| 60 | Thummler et al. | Vaccines | 2022 July | 4 | 1 | 3 | 8 |
| 61 | Tometten et al. | Journal of Infectious Diseases | 2022 July | 4 | 2 | 3 | 9 |
| 62 | Watcharananan et al. | American Journal of Transplantation | 2021 November | 4 | 1 | 3 | 8 |
| 63 | Westhoff et al. | Kidney International | 2021 September | 3 | 0 | 3 | 6 |
| 64 | Yahav et al. | Transplant International | 2021 November | 3 | 0 | 3 | 6 |
| 65 | Zhang et al. | Frontier in Immunology | 2022 September | 4 | 0 | 3 | 7 |
| 66 | Zhang et al. | Transplant Infectious Disease | 2021 November | 4 | 2 | 3 | 9 |

**Supplementary Figure S1**: Cellular immune response rate in SARS-CoV-2-naïve kidney transplant recipients after receiving different numbers of SARS-CoV-2 vaccine doses. (A) IFN-γ ELSIPOT assay. (B) IGRA. (C) Flow cytometric analysis.

**
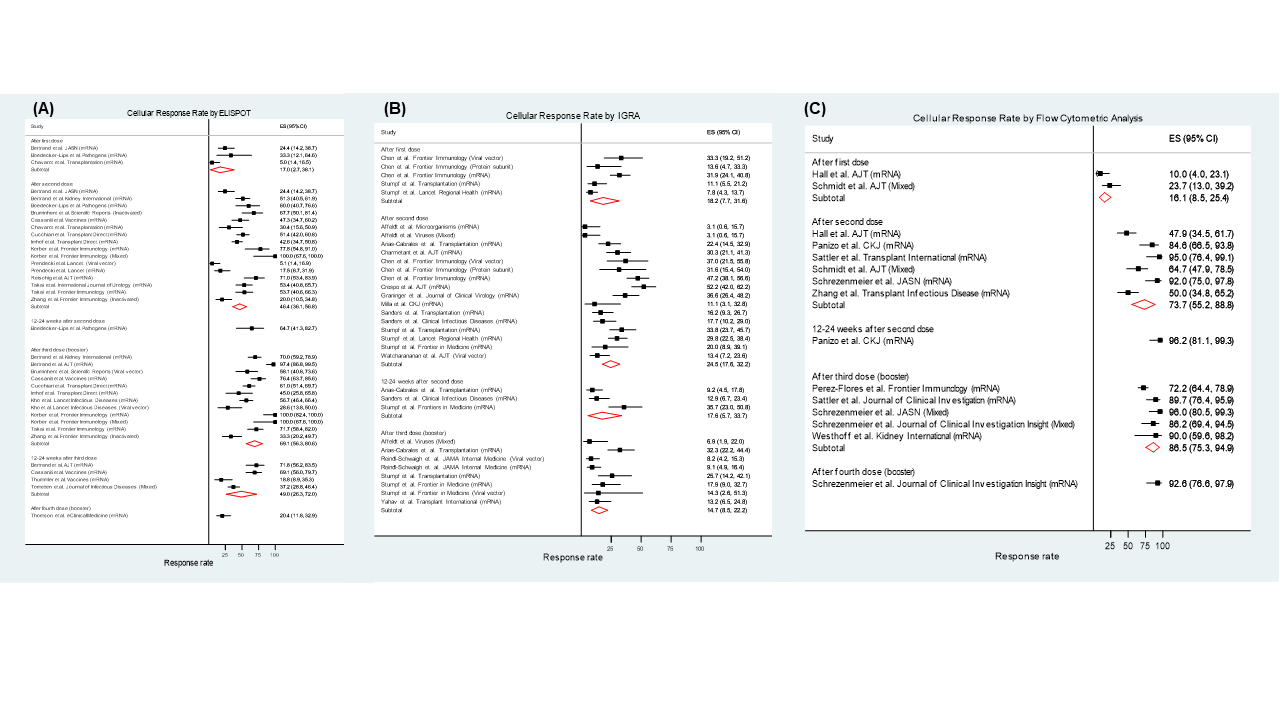
**

**Supplementary Figure S2**: Cellular immune response rate in kidney transplant recipients after receiving different numbers of SARS-CoV-2 vaccine doses, excluding studies that selected only non-seroconverters for the booster dose. (A) IFN-γ ELSIPOT assay. (B) IGRA. (C) Flow cytometric analysis.


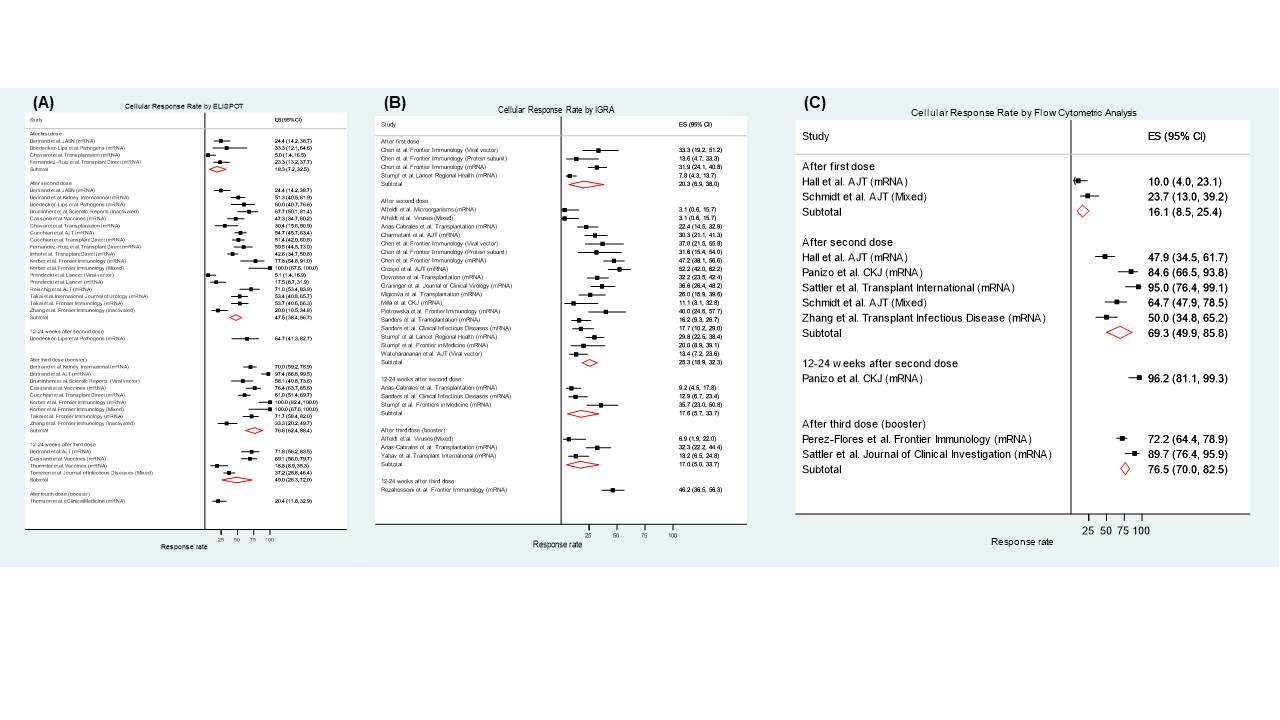


**Supplementary Figure S3**: Anti-spike protein antibody response rate in kidney transplant recipients, by timing of sample collection.

**Supplementary Figure S4:** Funnel plots of studies which compared overall cellular and antibody response rates.


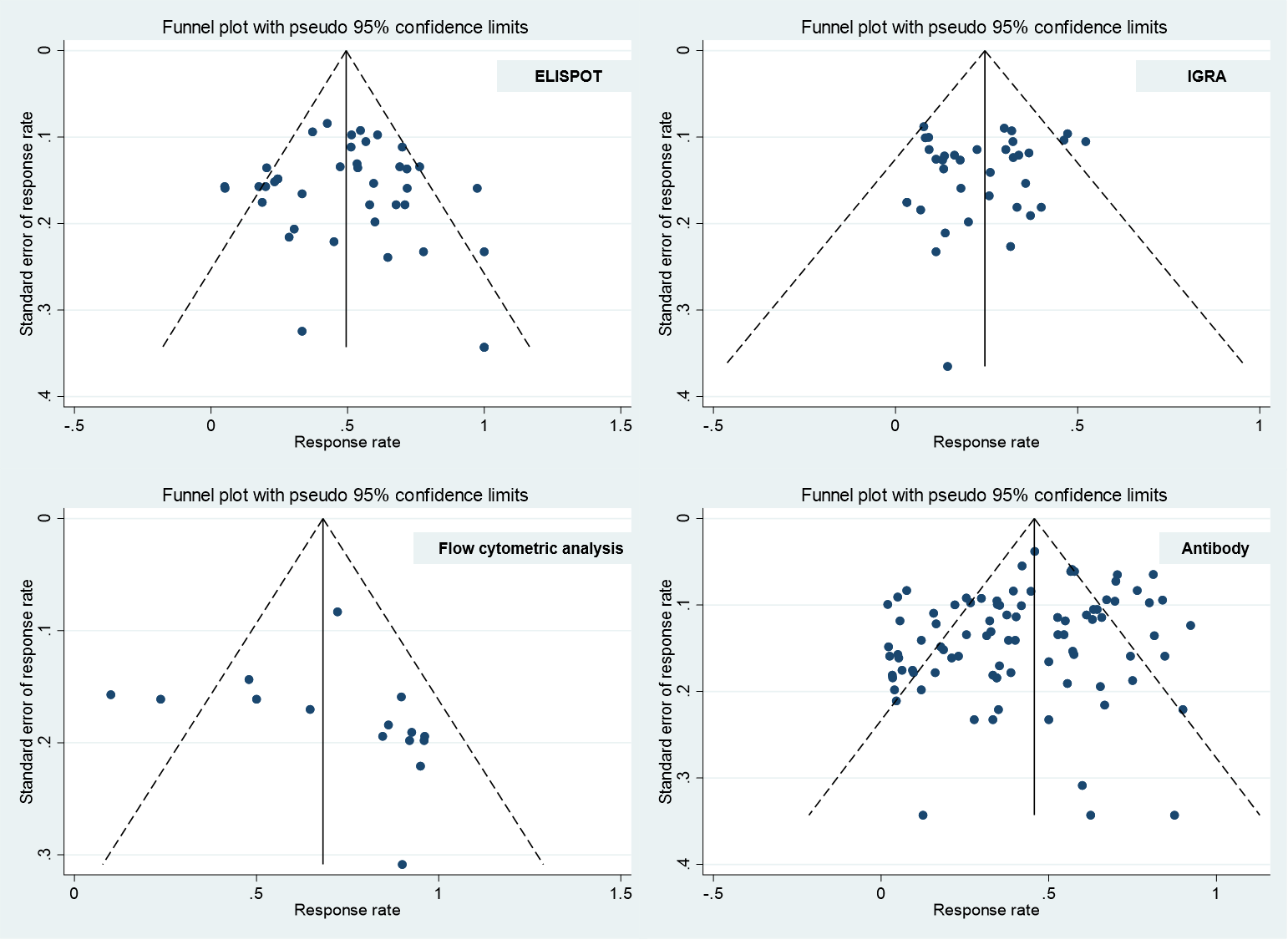


**
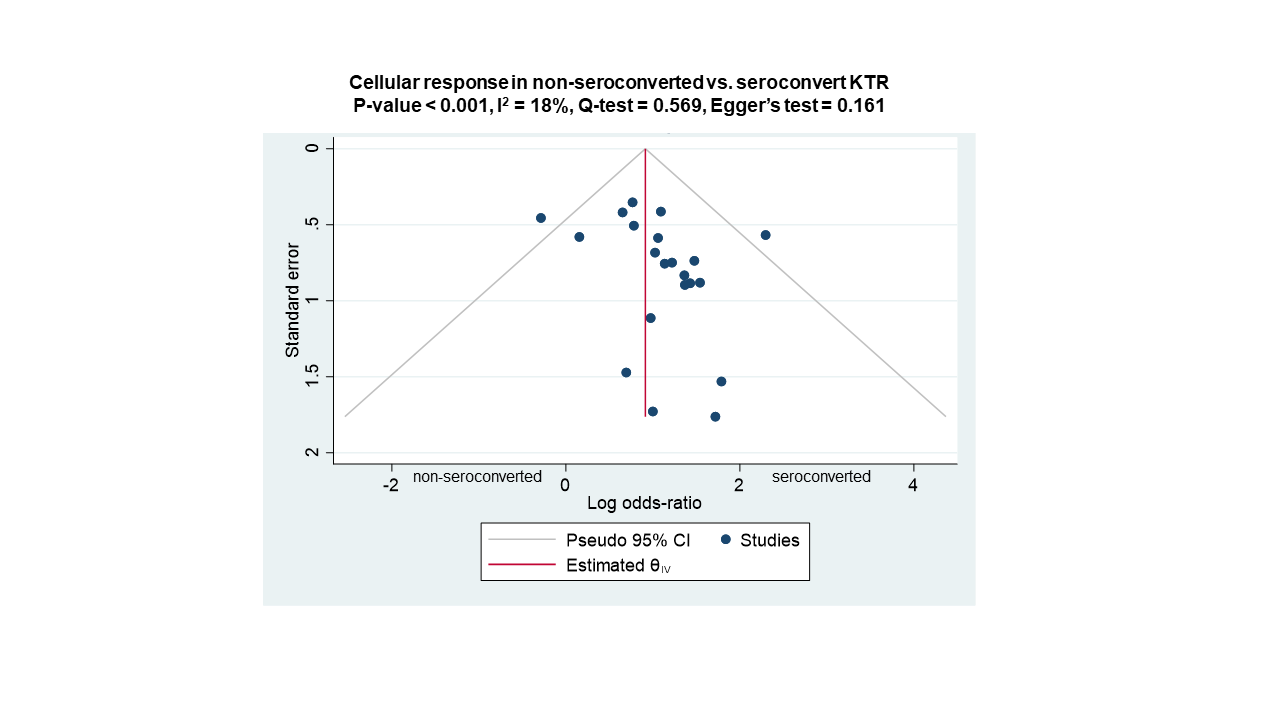
Supplementary Figure S5**: Funnel plot of studies which compared cellular immune responses in seroconverter vs. non-seroconverter kidney transplant recipients.

**
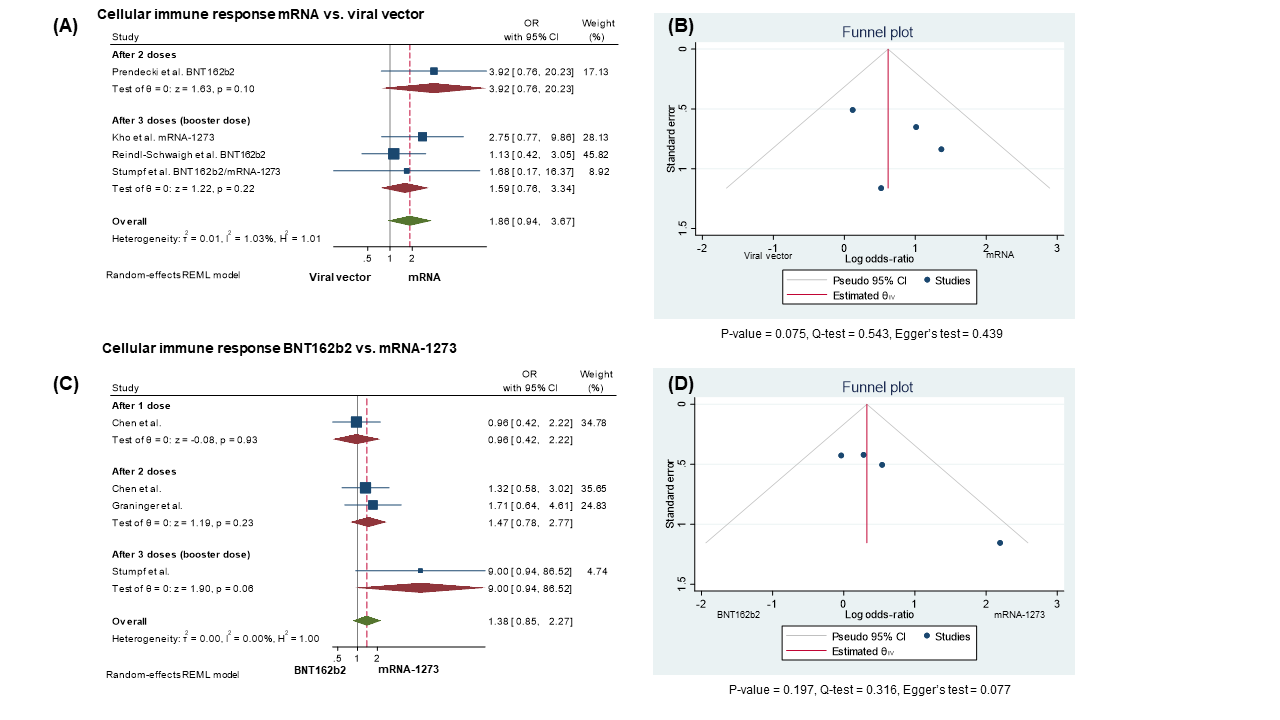
Supplementary Figure S6**: (A) A forest plot of cellular immune response in studies comparing mRNA vs. viral vector vaccine and (B) a funnel plot for these included studies. (C) A forest plot comparing cellular immune response in studies which compared BNT162b2 vs. mRNA-1273 and (D) a funnel plot for the included studies.

**Supplementary Figure S7**: Funnel plots for studies comparing cellular immune response rates in (A) kidney transplant recipients vs. dialysis patients and (B) kidney transplant recipients vs. control population.

**
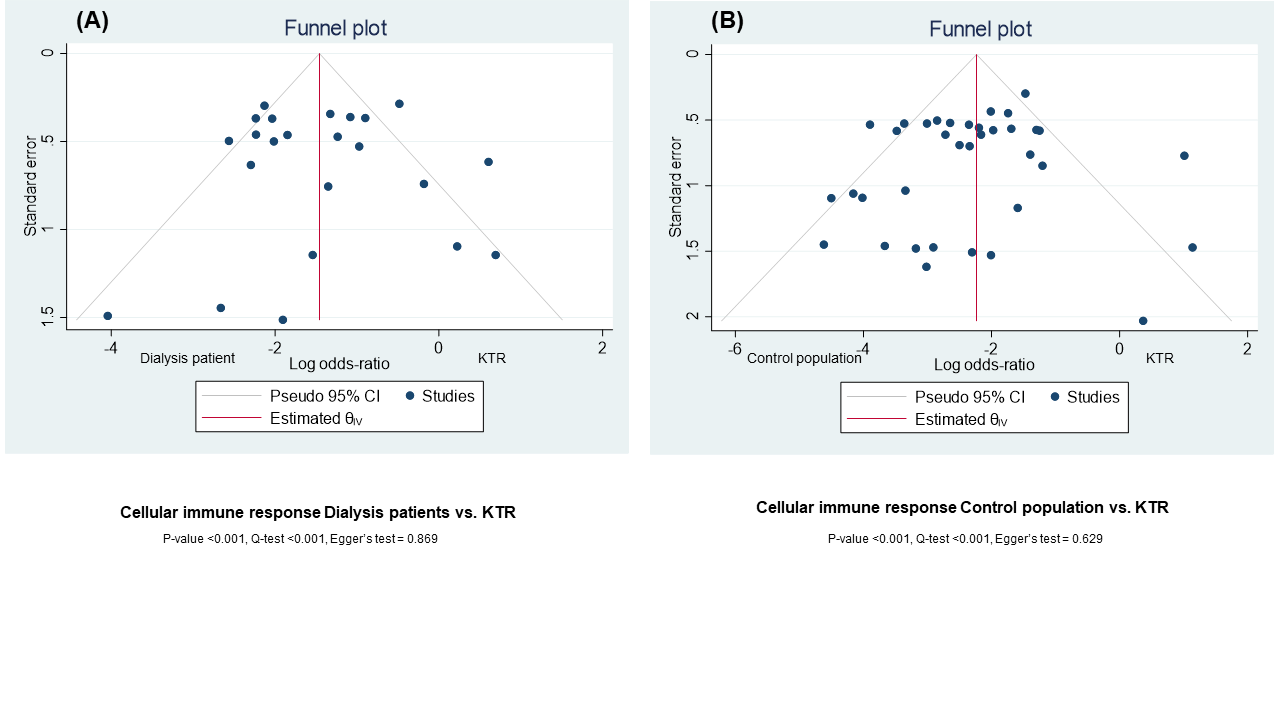
**


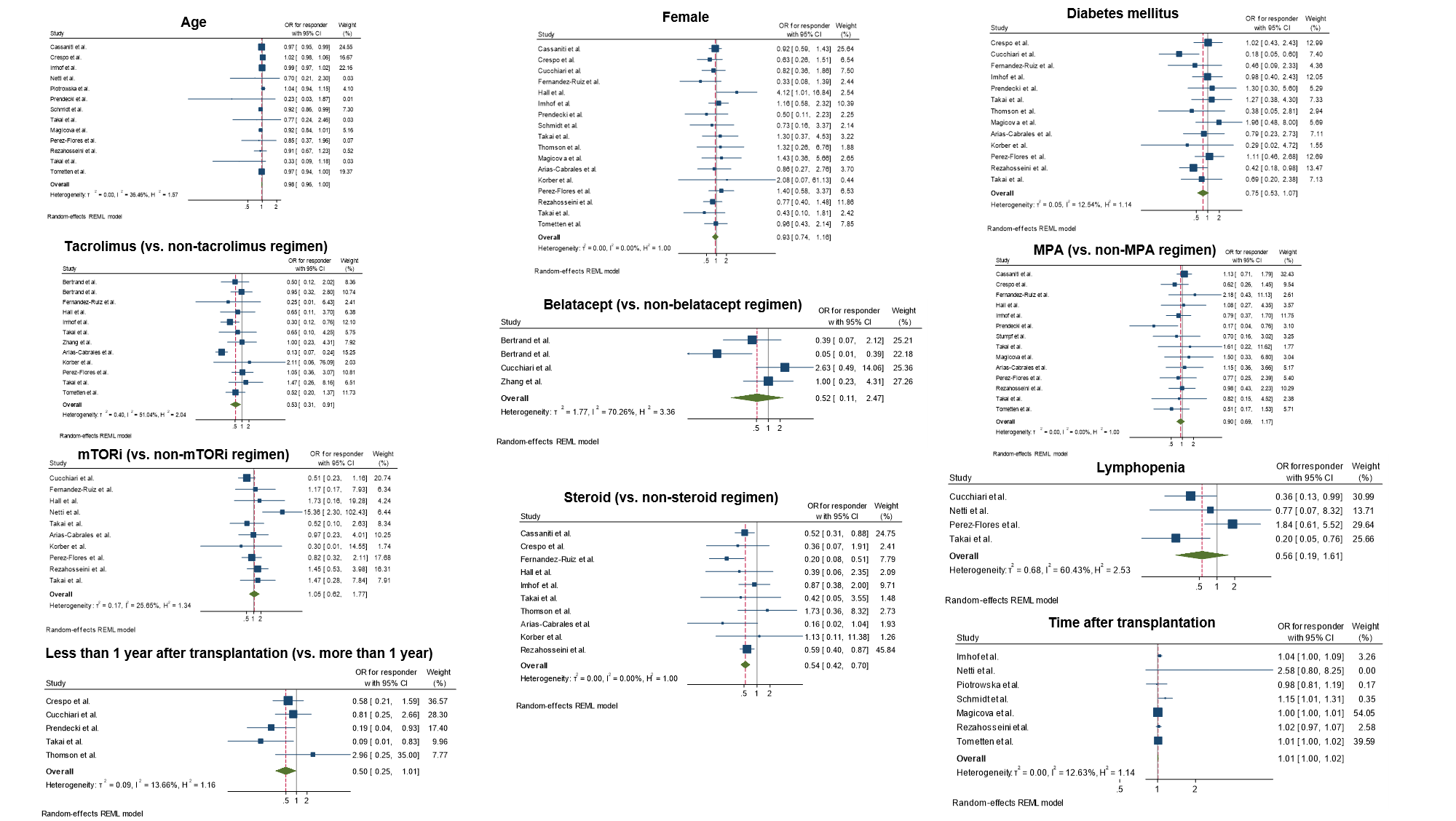
**Supplementary Figure S8**: Forest plots for studies including information on factors demonstrating an association with cellular immune response rates.

**Supplementary Figure S9**: Funnel plots for studies which compared factors demonstrating an association with cellular immune response rates.


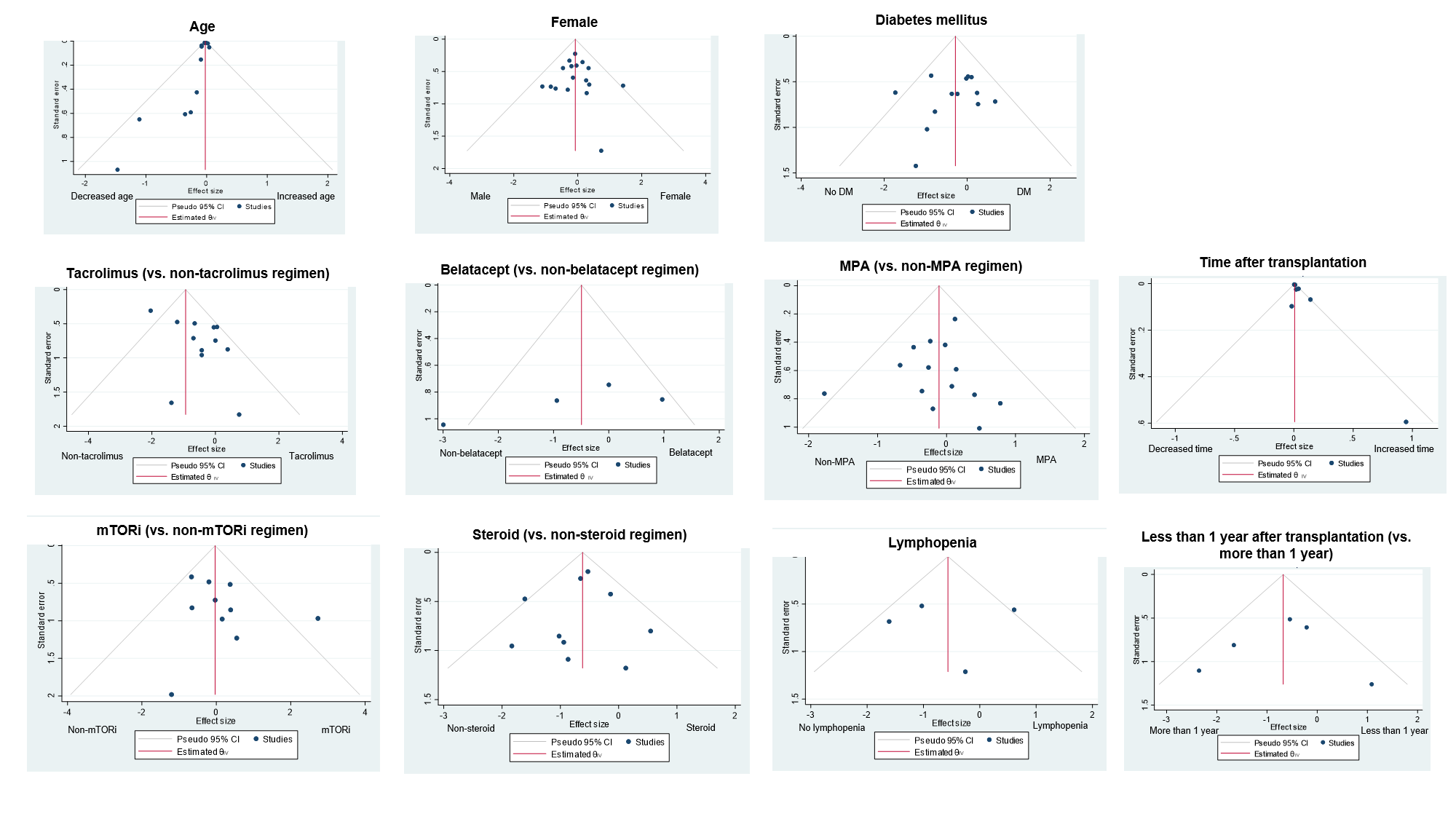

Supplement: Supplementary file 1 [file DataSheet_1.docx]
